# Successful heart transplantation as third cardiac operation in a 12 year-old Marfan patient

**DOI:** 10.1186/1749-8090-8-S1-O152

**Published:** 2013-09-11

**Authors:** M Pólos, T Hüttl, E Németh, O Balogh, B Ágg, K Benke, L Ablonczy, E Bartha, Z Szabolcs

**Affiliations:** 1 Heart and Vascular Center of Semmelweis University, Budapest, Hungary; 2 Department of Anesthesiology and Intensive Care, Semmelweis University, Budapest, Hungary; 3 National Heart Institute, Budapest, Hungary

## Background

Approximately 300.000 patients might be treated in Hungary because of heart failure. Out of this 900-1000 might be affected by end stage heart failure. These patients should be potentially treated by heart transplantation (HTX). The etiologic reasons of heart failure among transplanted patient are mainly cardiomyopathic or ischemic in origin (48%-44%). The valvular or congenital origin is rear (4%-2%). The prevalence of Marfan syndrome is 1-2/10.000; it means that we can calculate with 1000-2000 Marfan patients (MP) in Hungary. As it is mentioned in the literature MPs are often affected by a left ventricle dysfunction due to the characteristic Marfan cardiomyopathy, which may show a rapid progression in case of concomitant longstanding aortic or/and mitral regurgitation.

## Methods and results

Among the 243 patients underwent HTX in Hungary, there was only one Marfan patient. The 12 year-old male patient underwent previously a total aortic root replacement and after that a mitral valve implantation 4 and 2 years earlier. The symptoms of a left ventricle dysfunction were developed just a year after the second open heart procedure, but the progression was extremely fast resulting with a very severe heart failure. The MR imaging raised even the etiologic role of the Marfan cardiomyopathy in developing of this type of heart failure. The child, who was accepted to high urgent list of Eurotransplant got a donor heart after 31 days. After the operation he recovered uneventfully.

## Conclusion

Despite of the successful HTX, it remains controversial, how the longstanding immunosuppressive treatment will affect and damage later on the wall structure of the aorta (arch and the descendent) predisposing a later dissection of the descending aorta.

